# Fluorinated Polyethylene Propylene Ferroelectrets with an Air-Filled Concentric Tunnel Structure: Preparation, Characterization, and Application in Energy Harvesting

**DOI:** 10.3390/mi11121072

**Published:** 2020-12-01

**Authors:** Xi Zuo, Li Chen, Wenjun Pan, Xingchen Ma, Tongqing Yang, Xiaoqing Zhang

**Affiliations:** 1 Shanghai Key Laboratory of Special Artificial Microstructure Materials and Technology, School of Physics Science and Engineering, Tongji University, Shanghai 200092, China; 1832882@tongji.edu.cn (X.Z.); 18916269332@163.com (L.C.); wjpan_tj@163.com (W.P.); maxingchen@tongji.edu.cn (X.M.); 2 School of Materials Science and Engineering, Tongji University, Shanghai 200092, China

**Keywords:** ferroelectrets, longitudinal piezoelectric effect, radial piezoelectric effect, energy harvester, concentric tunnel structure, fluorinated polyethylene propylene

## Abstract

Fluorinated polyethylene propylene (FEP) bipolar ferroelectret films with a specifically designed concentric tunnel structure were prepared by means of rigid-template based thermoplastic molding and contact polarization. The properties of the fabricated films, including the piezoelectric response, mechanical property, and thermal stability, were characterized, and two kinds of energy harvesters based on such ferroelectret films, working in 33- and 31-modes respectively, were investigated. The results show that the FEP films exhibit significant longitudinal and radial piezoelectric activities, as well as superior thermal stability. A quasi-static piezoelectric *d*_33_ coefficient of up to 5300 pC/N was achieved for the FEP films, and a radial piezoelectric sensitivity of 40,000 pC/N was obtained in a circular film sample with a diameter of 30 mm. Such films were thermally stable at 120 °C after a reduction of 35%. Two types of vibrational energy harvesters working in 33-mode and 31-mode were subsequently designed. The results show that a power output of up to 1 mW was achieved in an energy harvester working in 33-mode at a resonance frequency of 210 Hz, referring to a seismic mass of 33.4 g and an acceleration of 1 *g* (*g* is the gravity of the earth). For a device working in 31-mode, a power output of 15 μW was obtained at a relatively low resonance frequency of 26 Hz and a light seismic mass of 1.9 g. Therefore, such concentric tunnel FEP ferroelectric films provide flexible options for designing vibrational energy harvesters working either in 33-mode or 31-mode to adapt to application environments.

## 1. Introduction

Recent decades have witnessed the increasing development of energy harvesting technology, which is driven by the demand for self-powered portable devices and wireless sensor network nodes in the Internet of Things (IoT) [1,2,3,4,5,6,7,8,9]. Energy harvesting refers to a renewable distributed energy technique converting ignored energy sources in environments, such as solar energy, heat gradient energy, and mechanical energy, into useful electrical energy. Among these energy sources, mechanical energy is available almost everywhere and can be harvested by means of electromagnetic, electrostatic, triboelectric, or piezoelectric transduction [10]. For the harvesters utilizing piezoelectric mechanisms, named piezoelectric harvesters (PEHs), one of the critical elements in the devices is the piezoelectric transducer, whose performance is strongly dependent on the properties of the piezoelectric materials [11]. Because of their small size, thin-film structure, high energy conversion efficiency, and durability, intensive studies have been conducted on PEHs as microelectromechanical system (MEMS) generators [12].

A variety of piezoelectric materials, including piezoelectric ceramics [13,14], ferroelectric polymers [15], and ferroelectrets [16], have been adopted in energy harvesting. Among them, the ferroelectret is a relatively new one that was first used in energy harvesters in 2012 [17]. However, owing to its specific features, such as strong piezoelectric effect, flexibility, environmental-friendliness, small acoustic impedance, easy processing, and low cost, more and more attention has been paid to ferroelectret-based energy harvesters in recent years [18].

Many kinds of polymers have been used to prepare ferroelectret films, including polypropylene (PP) [19], irradiation-crosslinked polypropylene (IXPP) [20], polytetrafluoroethylene (PTFE) [21], fluorinated polyethylene propylene (FEP) [22], cycloolefin copolymer (COC) [23], polyethylene-naphthalate (PEN) [24], cyclic transparent optical polymer (CYTOP) [25], polylactic acid (PLA) [26], etc. Considering energy harvesters are likely to work in harsh environments with high humidity and elevated temperatures, hydrophobic and thermally stable ferroelectrets are more favorable. Normally the hydrophobicity requirement can be met by packaging devices with waterproof plastics [27]. Therefore, it is apparent that thermal stability is a more critical issue in ferroelectrets. Relevant articles describe a significant improvement in thermal stability of FEP laminated samples compared to PP samples whose working temperature is normally less than 50 °C. These samples exhibit stable piezoelectric properties at 90 °C or higher [22,28].

In our prior work, we prepared laminated FEP ferroelectret films with an air-filled parallel tunnel structure exhibiting strong longitudinal and transverse piezoelectric effects. Furthermore, the vibrational energy harvesters based on such material can generate high power when working in 31-mode [22,29,30]. Being an extension of this concept, FEP ferroelectret films with a concentric tunnel structure were designed and prepared in this study. The fabricated films display similar properties as parallel tunnel samples with remarkable longitudinal and radial piezoelectric activities [29,31]. However, differing from parallel tunnel FEP ferroelectret films, the presently studied samples can be subjected to stresses on a two-dimensional plane as when working in 31-mode, which may increase flexibility in device design. Because of their radially symmetrical structure, concentric tunnel FEP ferroelectret films could be a more promising option for piezoelectric circular diaphragm (PCD) harvesters, which feature greater response to small strains and higher output power than cantilever beam harvesters. In this article, we report the preparation and characterization of concentric tunnel FEP ferroelectret films, and explore their applications in vibrational energy harvesters working in 33- and 31-modes.

## 2. Preparation and Characterization of Concentric Tunnel FEP Films

### 2.1. Preparation Procedure

**Figure 1:**
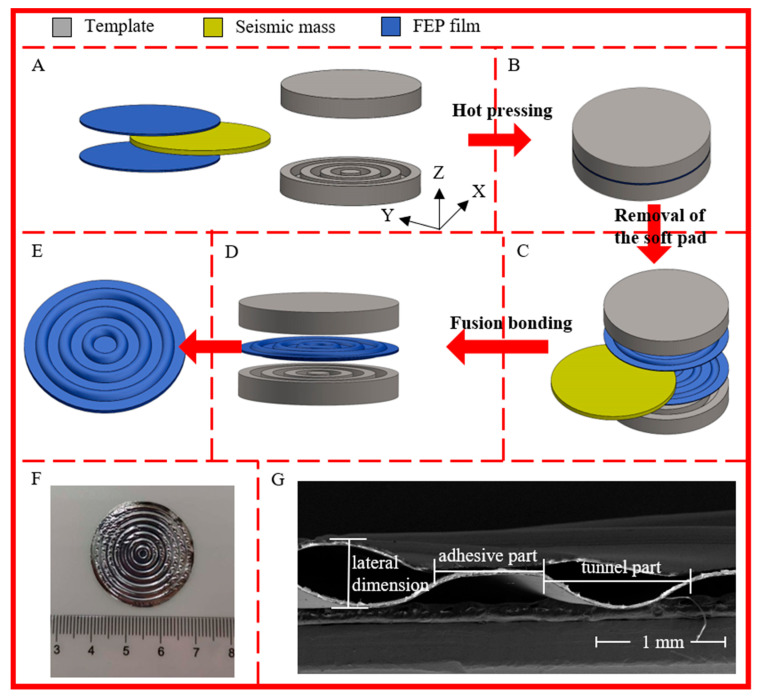
Schematic of fabrication and images of the concentric tunnel fluorinated polyethylene propylene (FEP) films. (**A**) Stacking sequence. (**B**–**D**) are the steps of thermoplastic molding. (**E**–**G**) are the schematic model, optical image, and cross-sectional SEM image of concentric tunnel FEP films, respectively.

The 12.5 µm thick FEP films used in this study were supplied by DuPont. The preparation procedure of concentric tunnel films is schematically shown in Figure 1. Firstly, a pair of rigid circular templates with multiple homocentric annular ‘ditches’ were made. The ditches had a width of 1.5 mm and a depth of 0.5 mm. The distance between the adjacent ditches was 0.5 mm. In the preparation process, two FEP films and a soft rubber pad were first sandwiched between two templates in sequence as shown in Figure 1A. The soft rubber pad served as a buffer during the patterning step. Then, the films were patterned at a temperature of 40 °C and a pressure of 2 MPa for 4 min (Figure 1B). These parameters are used for obtaining a plastic deformation in FEP films at a relatively low temperature with the help of pressure. After that, the soft pad was removed, two layers of films were buckled together with the templates (Figure 1C), and the stacks were heated in a muffle furnace for 13 min at 320 °C. [22] After cooling at room temperature, fusion bonding of the two patterned FEP films was achieved, thus obtaining a film with an air-filled concentric tunnel structure (Figure 1D,E). Figure 1F and 1G show an optical image and cross-sectional scanning electron microscope (SEM) image of the fabricated concentric tunnel FEP films, respectively.

As shown in Figure 1G, owing to the special tunnel structure, the samples were divided into the bonded parts and the air-filled tunnel parts, whose thickness was obviously uneven. The minimum thickness, *t*_min_, which referred to the bonded parts of the two layers of FEP films, was assumed to be 25 µm. At the same time, with a pressure of 0.36 kPa applied on the entire upper surface by the F-55 thickness gauge, the maximum thickness, *t*_max_ corresponding to the center of the tunnel area was measured as approximately 340 μm for the samples.

**Figure 2:**
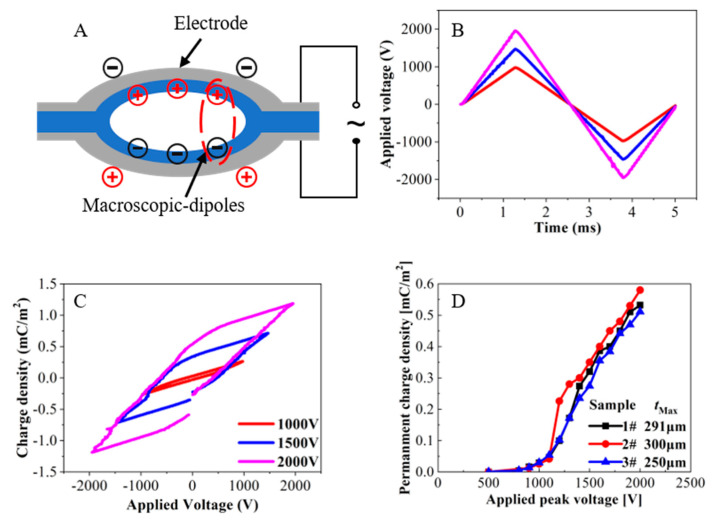
Contact polarization of the concentric tunnel FEP films. (**A**) Schematic of contact polarization. (**B**) Triangular voltage applied to the films. (**C**) Hysteresis loops of an FEP sample polarized by different applied peak voltages. (**D**) Permanent charge density on the external electrode as a function of the applied peak voltage.

In the following charging step, the concentric tunnel FEP films were first metallized with Al on both surfaces. Then, the internal charging of the tunnels was achieved by contact charging in air with triangular voltages as shown in Figure 2A. The triangular voltages were provided by a ferroelectric analyzer (Radiant Precision Multiferroic II), and their periods in the present case were 5 ms, as seen in Figure 2B. Figure 2C shows hysteresis loops of an FEP sample polarized by various peak voltages. The voltages were applied for many cycles, and always terminated electronically at zero to achieve the maximum polarization. Previous work indicated that the permanent charge density, which is corresponds to the induced charge density on the external electrode, is a function of the applied peak voltage [32]. As shown in Figure 2D, the charge density reached about 0.55 mC/m^2^ at a peak voltage of 2 kV.

### 2.2. Piezoelectric Response

The piezoelectric coefficient is an important parameter reflecting the coupling effect between mechanical and electric quantities of piezoelectric materials. In the present work, three methods, namely quasi-static, dynamic, and acoustic methods, were utilized to characterize the piezoelectric properties in the concentric tunnel FEP films. The longitudinal piezoelectric coefficient *d*_33_ can be directly determined by the following equation [33]:

(1) d33=QF

where *Q* and *F* are the induced charges on the surface electrodes and the force applied to the sample, respectively. Although it is complicated to directly measure the radial piezoelectric coefficient, *d*_31_, of the sample with such a complex structure, we propose a radial sensitivity, *M_r_*, being relative to the location of the force applied, representing the piezoelectric effect in the radial direction of the ferroelectret samples. In this study, the force is applied to the centermost area of the sample. Thus, the *M_r_* can also be derived from

(2) Mr=QF

#### 2.2.1. Measurement of Piezoelectric Responses by Quasi-Static Method

Using the quasi-static method, as a certain external mechanical force, *F*, was applied to or removed from the sample in the z-direction, the induced charge *Q* generated on the surface electrodes was recorded by an electrometer (Keithley 6514), thereby directly calculating *d*_33_ and *M_r_* following Equations (1) and (2). The quasi-static method was also used to reflect the pressure dependence of the piezoelectric activity in the concentric tunnel FEP films.

**Figure 3:**
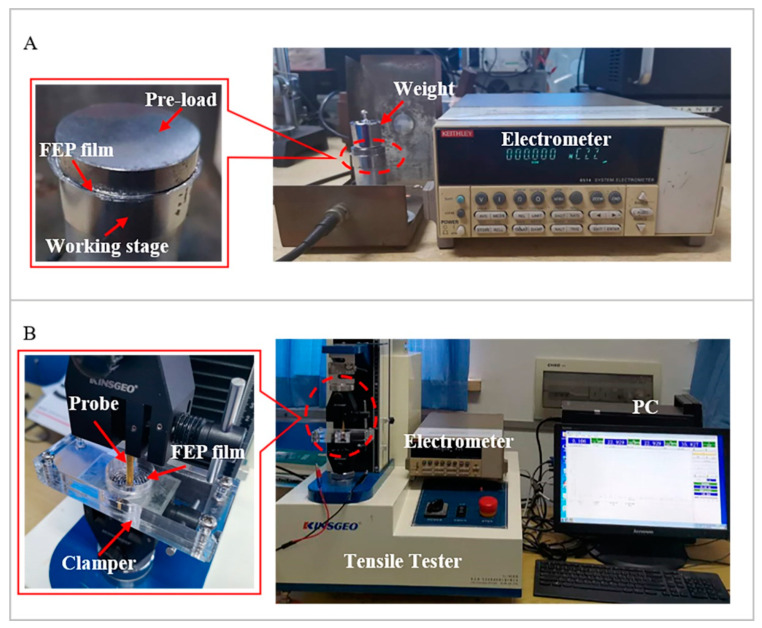
(**A**) Setup for measurement of the quasi-static coefficient, *d*_33_, in concentric tunnel FEP films. (**B**) Setup for measurement of quasi-static radial piezoelectric sensitivity, *M_r_*.

In order to measure the quasi-static coefficient *d*_33_, the sample was sandwiched between the working stage and a circular metal plate (see Figure 3A). The plate had the same diameter as the sample (30 mm) and served as a static pre-load to ensure that the tunnels were slightly deformed to avoid a bending effect. The force was provided by a series of weights ranging from 10 to 200 g. Since in practice, removal of weights was much more easily controlled than their application, the weights were placed on the plate for a relatively long time and then rapidly removed. The induced charge, *Q*, integrated over 10 s after weight removal was recorded by an electrometer.

Figure 3B shows the measuring setup for the quasi-static radial piezoelectric sensitivity, *M_r_*. In this case, the edge of the sample was clamped, while the center part was free. The sample was stretched radially with a static force, *F*, applied at its center by a tensile tester (KJ-1065A) equipped with a cylindrical probe. The diameter of the probe was 2 mm, which was the same size as that of the centermost circular bonded part. When the static force was applied to the sample, all tunnels were stretched in the radial direction. The induced charge was recorded by the electrometer after the removal of the force.

#### 2.2.2. Pressure Dependence

**Figure 4:**
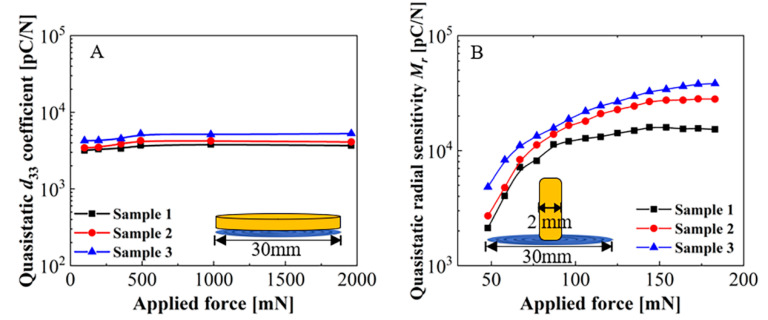
Pressure dependence of the concentric tunnel FEP films with diameters of 30 mm. (**A**) Quasi-static piezoelectric coefficients, *d*_33_, as a function of applied force. (**B**) Quasi-static radial sensitivity, *M_r_*, as a function of applied force on the centermost area for FEP samples.

As mentioned above, the quasi-static longitudinal and radial piezoelectric responses were measured to investigate the pressure dependence of the concentric tunnel FEP films. Results on the relation between the quasi-static piezoelectric coefficient *d*_33_ and the applied pressure for three FEP film samples are illustrated in Figure 4A. This figure indicates the example of sample 3, marked in blue, in a small pressure range from 100 to 1960 mN. The *d*_33_ coefficients increased steadily from 4261 pC/N at 100 mN to 5233 pC/N at 490 mN. As the force continued to increase, the values started to level off and then remained at 5280 pC/N within a higher-pressure range of 490 mN to 1960 mN. The results indicate that the samples had higher longitudinal piezoelectric activity at a relatively low pressure of about 490 mN.

The results of the quasi-static radial piezoelectric sensitivity, *M_r_*, indirectly reveal the pressure dependence of the radial piezoelectric activity. As shown in Figure 4B, three typical samples were tested under the same conditions. Taking sample 3 as an example, one observes that the *M_r_* increases sharply from 4820 pC/N to 18,767 pC/N at relatively small static forces ranging from 50 to 100 mN, while the rate of increase then slows down with the force increasing from 100 to 200 mN. The values reach up to around 40,000 pC/N at 200 mN. Despite the inconsistent behaviors of the samples, which is due to inevitable structural differences during the preparation process under the given experimental conditions in our lab, the results have import for determining the order of magnitude. The large radially piezoelectric sensitivity must be attributed to the significant amplification of applied force in 31-mode [22]. More work is underway to improve the uniformity of the samples.

#### 2.2.3. Measurement of Piezoelectric Responses by Dynamic Method

The subsequent dynamic measurements showed the frequency dependence of the concentric tunnel FEP films and helped to assess their potential applicability at different frequencies. The vibration system for dynamic measurement consisted of a digital audio analyzer (dScope Series III), a vibration exciter (B&K, Type 4809), a power amplifier (B&K, Type 2713), a charge amplifier (B&K, Type 2635), and an accelerometer (B&K 4382). The acceleration of the system was provided by the exciter, which was driven by the signal generated by the digital audio analyzer by virtue of the power amplifier. The charge generated on the electrode in short circuit was amplified by the charge amplifier, collected by the digital audio analyzer, and recorded by a computer, and the applied acceleration, *a*, was measured by the accelerometer.

**Figure 5:**
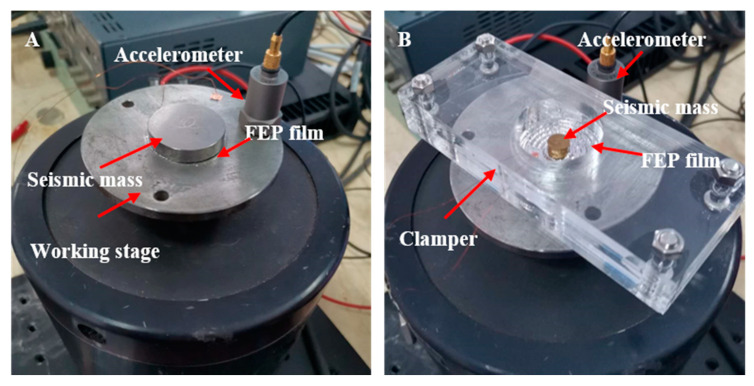
(**A**) Setup for measurement of the dynamic coefficient, *d*_33_, of the concentric tunnel FEP films. (**B**) Setup for measurement of the dynamic radial piezoelectric sensitivity, *M_r_*.

The dynamic *d*_33_ coefficient was measured with the setup shown in Figure 5A. The loaded forces on the sample were composed of a static force of *mg* and a dynamic force of *ma* [34]. A pre-load with a diameter of 30 mm and a mass of 30 g was placed directly at the top of the sample without adhesion. Thus, during vibration, the acceleration had to be limited to 2.7 m/s^2^ or less to avoid the sliding of the seismic mass. Meanwhile, the experimental setup for dynamic sensitivity, *M_r_*, is depicted in Figure 5B. The seismic mass placed at the center part had a diameter of 8 mm and a weight of 1.2 g.

#### 2.2.4. Frequency Dependence

**Figure 6:**
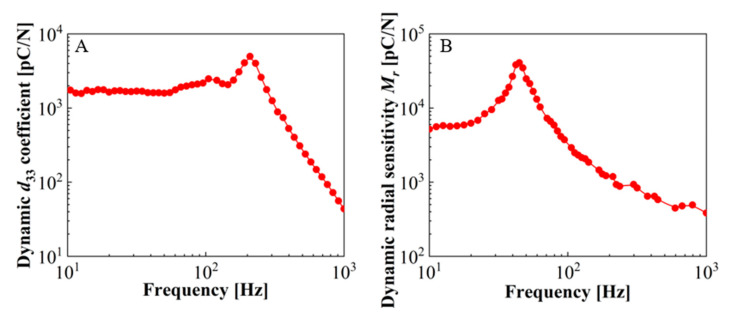
Frequency dependence of the concentric tunnel FEP films. (**A**) Dynamic piezoelectric coefficient, *d*_33_, as a function of frequency. (**B**) Dynamic piezoelectric radial sensitivity, *M_r_*, as a function of frequency.

Results on the dynamic piezoelectric responses are illustrated in Figure 6. The results not only indicate the frequency dependence of the longitudinal and radial piezoelectric responses, but also the information of the resonance frequency of 33-mode and 31-mode energy harvesters.

The resulting frequency dependence of the *d*_33_ coefficient for a representative sample with a quasi-static *d*_33_ coefficient of 4346 pC/N is shown in Figure 6A. The experimental frequency ranged from 10 to 1000 Hz, and the seismic mass was 30 g. As the frequency of the vibration system increased, the dynamic *d*_33_ was stabilized over a low-frequency range of 10 to 150 Hz around 1700 pC/N. The piezoelectric response then reached a peak of 4979 pC/N at the resonance around 210 Hz, followed by a dramatic drop as expected. The dynamic radial sensitivity *M_r_* of the same sample with a seismic mass of 1.2 g is depicted in Figure 6B. The dynamic *M_r_* is calculated as 40,900 pC/N at the resonance frequency of about 45 Hz. The radial piezoelectric activity of the sample also remained at a high level at resonance, but decreased significantly with the increase of the frequency above resonance.

#### 2.2.5. Measurement of d_33_ by Acoustic Method

**Figure 7:**
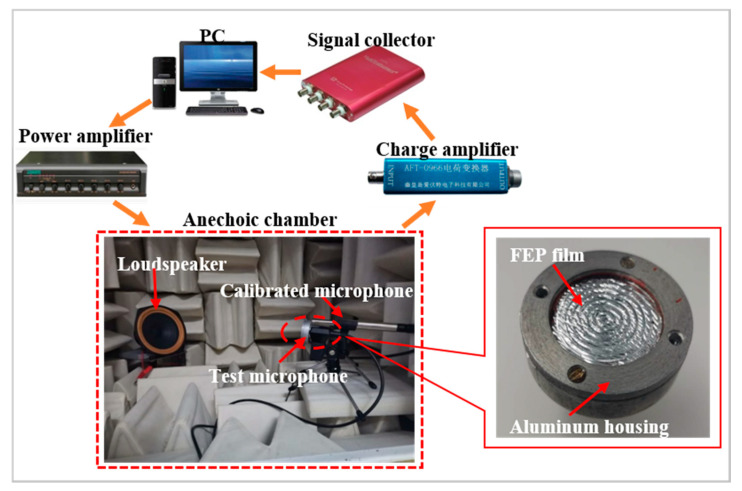
Setup for measurement of the dynamic coefficient *d*_33_ of the concentric tunnel FEP films by the acoustic method.

In this work, the acoustic method was also carried out to determine the dynamic coefficient *d*_33_ (see Figure 7) [32]. The advantage of this method is that although the surface is uneven, the dynamic coefficient *d*_33_ can be determined as an average value by applying a free-field sound pressure, *p*, on the sample. The 30 mm diameter test microphone consisted of the sample and an Al housing, which was utilized to ensure good shielding for acoustic measurements. The sound source was provided by a computer-controlled dynamic loudspeaker with a 300–1000 Hz broadband response and was amplified simultaneously by a power amplifier (DSPPA MP200PIII). The induced charge, *Q*, was amplified with a charge amplifier (AFT-0966) and then recorded by a signal collector (MI, VT DSO-2820). In this case, the loudspeaker, the test microphone together with a calibrated quarter-inch microphone (KW-4420), which was used to determine the reference free-field sound pressure *p*, were all located in an anechoic chamber. The test microphone and the calibrated microphone were placed equidistantly on both sides along the center axis 30 cm from the loudspeaker.

Therefore, the pressure sensitivity, *M_p_*, of the test microphone was obtained from the calibrated microphone using a replacement method as follows [35,36]:

(3) Mp=Vp

where *V* is the output voltage generated by the microphone, and the dynamic coefficient *d*_33_ was obtained by means of

(4) d33=Mp·CA

where *C* is the capacitance and *A* is the area of the sample.

#### 2.2.6. Acoustic Response

**Figure 8:**
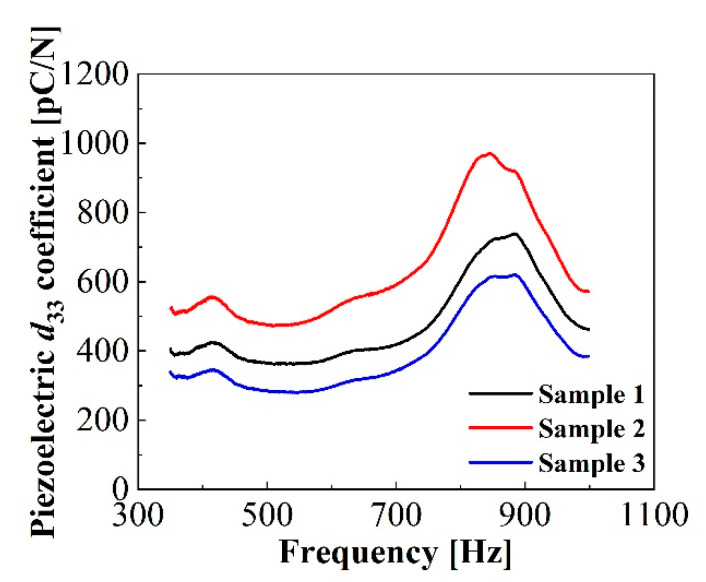
Acoustic dependences of the concentric tunnel FEP films with diameters of 30 mm.

Results of the dynamic coefficients *d*_33_ calculated by the acoustic method following Equations (3) and (4) are presented in Figure 8. For the typical samples, dynamic coefficients *d*_33_ obtained acoustically were between 300 and 500 pC/N at 350 Hz. The acoustic values were much smaller than that determined by the dynamic method, which was calculated as 800 pC/N at 350 Hz. This must be due to the fact that only very sensitive protruding tunnel parts were excited in the dynamic measurement using a shaker, while the whole sample, including piezoelectrically active tunnel parts and inactive fusion bonding parts, was exposed to the acoustic pressure. Therefore, the averaging piezoelectric coefficients *d*_33_ obtained acoustically under uniform acoustic waves are smaller than that obtained by the dynamic method [28]. In addition, there exist two response peaks at frequencies of 415 Hz and 860 Hz, respectively. The resonance peak at 415 Hz may be related to the bending extension in the radial direction under the sound pressure, and the peak at 860 Hz is associated with the well-known diffraction of the acoustic wave in acoustic measurements, which happens when the size of the test microphone is comparable or larger than the square of the wavelength [32].

### 2.3. Dielectric Resonance Spectrum

The dielectric resonance spectrum (DRS) was utilized to obtain the electromechanical properties of the concentric tunnel FEP films. Under the two-sided free boundary condition, the measurement was carried out by a precision impedance analyzer (Agilent 4294A). Due to their remarkable radial and longitudinal piezoelectric activities, the radial extension and the thickness extension of samples were visible, which were marked by RE mode and TE mode [37,38]. The RE mode referred to the bending extension in the radial direction, while the TE mode referred to the deformation in the thickness direction when the sample was pressed.

Young’s Modulus for the specimen in the thickness direction (*Y_t_*) can be determined by

(5) $$Y_{t}=4f_{a}{}^{2}t\rho_{s}$$

where *f_a_* is the anti-resonance of TE mode, *t* the thickness of the film, and *ρ_s_* the area density. And Young’s Modulus in the radial direction (*Y_r_*) is given by

(6) $$Y_{r}={\left(\frac{2\pi rf_{a}}{2.08}\right)}^{2}\left(1-\nu^{2}\right)\frac{\rho_{s}}{t}$$

where ν is Poisson’s ratio. For foam and polymer, the Poisson’s ratio is generally between −0.7 to 0.5 [39]. Unlike the re-entrant structure materials exhibiting a negative Poisson’s ratio [40], the value of the concentric tunnel FEP ferroelectrets ranges from 0 to 0.5. As mentioned above, the thickness of the sample is not uniform; therefore, the lower and upper limits of Young’s Modulus were calculated from *t*_max_ and *t*_min_ to obtain *Y*_max_ and *Y*_min_, respectively [41]. The actual values of *Y_t_* and *Y_r_* for the concentric tunnel FEP samples must be in between the corresponding values.

**Figure 9:**
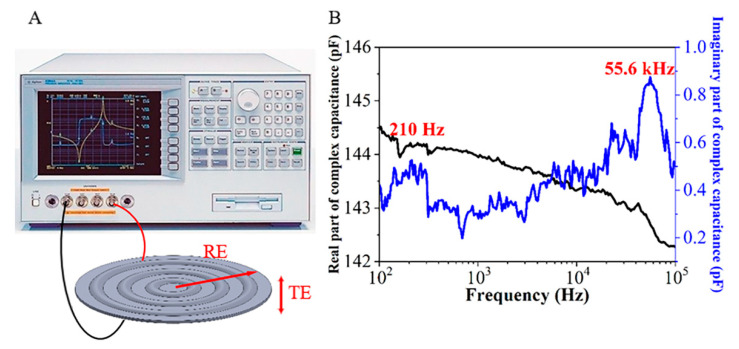
Dielectric responses of a concentric tunnel FEP sample with a diameter of 30 mm. (**A**) Measurement setup. (**B**) Dielectric resonance spectrum (DRS) of a concentric tunnel FEP sample.

The measuring setup of DRS for the concentric tunnel FEP film sample is shown in Figure 9A. Results of complex capacitance as a function of frequency ranging from 0.1 to 100 kHz for an FEP film sample clearly point out RE and TE modes and corresponding loss peaks at 210 Hz and 55.6 kHz, respectively (see Figure 9B). For all tested samples, the TE mode has a resonance in a frequency range of 50 to 60 kHz, which is in agreement with the measurement result of the parallel tunnel samples in [22]. According to Equation (5), taking the maximum and minimum thickness—*t*_max_ = 340 μm and *t*_min_ = 25 μm—of the samples quoted above, and the area density of 0.16 kg/m^2^, the maximum and minimum limits of the Young’s Modulus in the thickness direction for the specimen were determined as *Y_t_*_-max_ = 0.44 MPa and *Y_t_*_-min_ = 0.04 MPa, respectively, while the resonance frequencies of RE mode were measured as approximately 200 to 500 Hz, which are lower than those of parallel tunnel samples (at 1.8–6.5 kHz) [22]. Together with the Poisson’s ratio ranging from 0 to 0.5, the results were used to calculate the Young’s Modulus in the radial direction with Equation (6), giving the calculated values of *Y_r_*_-max_ = 45 MPa and *Y_r_*_-min_ = 0.46 MPa. The actual values of *Y_t_* and *Y_r_* for the concentric tunnel FEP films must be in between.

On the other hand, within the measurement frequency range, the real part of the capacitance is about 140 pF and the imaginary part is less than 1 pF. The real part is more than three orders of magnitude higher than the imaginary part. Therefore, the loss of circular tunnel FEP films is considered relatively small, owing to the extremely small conductivity in the FEP ferroelectrets.

### 2.4. Measurements of Thermal Stability

To evaluate the performance of the concentric tunnel FEP films at elevated temperatures, the isothermal decay of *d*_33_ coefficients at various temperatures and short-circuit thermally stimulated discharge (TSD) current spectra were measured. Firstly, the measurement of the isothermal decay of *d*_33_ coefficients was conducted. The tested samples were prepared under the same conditions and polarized at room temperature, but stored at room temperature (RT), 90 °C, or 120 °C for more than 20 h. The curves of the normalized *d*_33_ coefficient were obtained using a quasi-static method.

**Figure 10:**
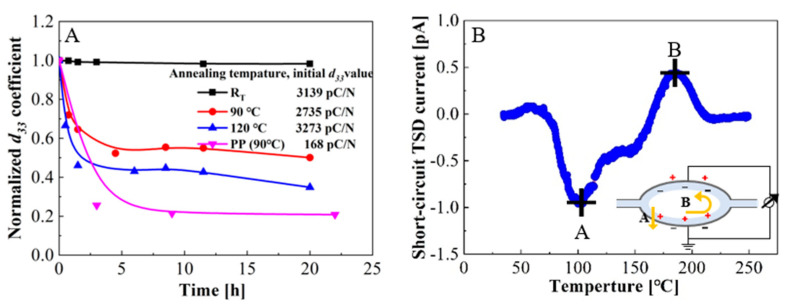
Thermal stability of the concentric tunnel FEP films. (**A**) Normalized *d_33_* coefficients’ isothermal decay. (**B**) Short-circuit thermally stimulated discharge (TSD) current spectrum.

Figure 10A shows the isothermal decay curves of the *d*_33_ coefficients of the concentric tunnel FEP films. The initial *d*_33_ coefficients of the samples at room temperature (RT), 90 °C, and 120 °C were 3139, 2735, and 3273 pC/N, respectively. The samples stored at 90 °C and 120 °C decreased significantly in the first two hours and were basically stable after 6.5 h. After 20 h annealing, *d*_33_ coefficients dropped to 3076, 1367, and 982 pC/N, corresponding to about 98, 50, and 35% of the initial values, respectively. Compared with the thermal stability of PP ferroelectret films [28], which retain only 20% of the initial value after annealing at 90 °C for 20 h, concentric tunnel FEP ferroelectret films exhibit higher thermal stability, thus ensuring their higher operating temperature. This is because the stability of space charges in FEP is much better than in PP. Besides, the experimental results are in agreement with those of the parallel tunnel FEP films, which prove that the laminated two-layer FEP films with an air-filled tunnel structure have potential applicability at elevated working temperatures [41].

For further study of the thermal stability of charges and distribution of charge traps in the concentric tunnel FEP films, the TSD current spectra were conducted in a short-circuit arrangement [28,42]. The polarized samples were heated in a temperature-controlled chamber where the temperature rose from 30 to 250 °C at a rate of 3 °C/min, and the generated current was recorded with an electrometer (Keithley 6514) simultaneously. It is physically impossible to distinguish the contribution of two polar carrier signs. Hence, the short-circuit TSD currents provided information on the overall charge transport processes during discharge.

As shown in Figure 10B, the short-circuit TSD current spectrum comprises a negative peak at 100 °C and a positive peak at 185 °C. The negative current peak indicates that the positive charges mainly drift through the solid dielectric layer via path A shown in the inset. When the temperature reached 185 °C, the positive current reached the maximum, revealing that detrapped positive charges favorably moved along the inner surface through path B and were compensated with negative charges. These results are in agreement with those published previously [41].

## 3. Fabrication and Performance Assessment of Energy Harvesters

### 3.1. Fabrication of Energy Harvesters

Combined with the measurement results of the piezoelectric responses introduced above, two energy harvesters, working in 33- or 31-mode, were designed and fabricated to maximize the utilization of their prominent longitudinal and radial piezoelectric responses. Then, the feasibility of concentric tunnel FEP ferroelectrets serving as the transduction material in energy harvesters was evaluated.

**Figure 11:**
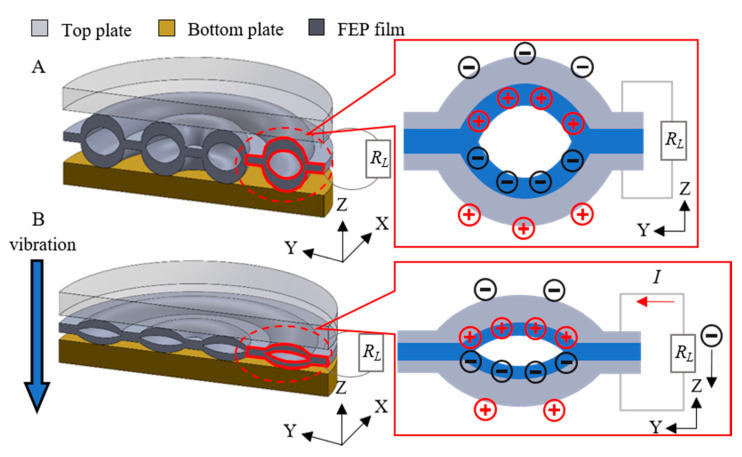
Schematic model and working mechanism of the 33-mode energy harvester. (**A**) The initial state of the 33-mode energy harvester. (**B**) The pressed state of the 33-mode energy harvester.

The design and the schematic of the working principle of the 33-mode energy harvester are indicated in Figure 11. The 33-mode energy harvester consisted of a top plate, a bottom plate, and an FEP film. For the sake of restricting the deformation of the film in all directions except vertically, the film was fixed on the bottom plate. Hence, the film remained flat during vibration to avoid the effect of bending deformation. The top plate with the same diameter as the film was used as the pre-load. When an external force was applied on the device, the top plate helped distribute the force evenly to each tunnel, thus deforming the tunnels. During vibration, sinusoidal variations of the air-gap thickness led to the change of induced charge density and thus current, *I*, in the external circuit.

**Figure 12:**
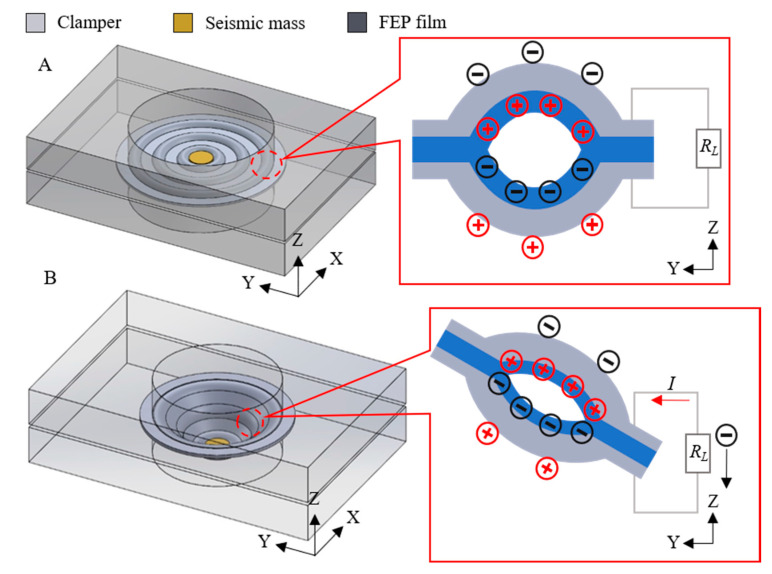
Schematic model and working mechanism of the 31-mode energy harvester. (**A**) The initial state of the 31-mode energy harvester. (**B**) The deflected state of the 31-mode energy harvester.

The 31-mode energy harvester was designed as having the boundary region fixed and the center part free (see Figure 12). The clampers were made of acrylic plates with a circular hole in the center with the same diameter as the samples. When the seismic mass was placed in the center, the tunnels were stretched radially in response to the applied force during vibration, resulting in changes of the dipole moments.

### 3.2. Performance Assessment of Energy Harvesters

Finally, the performance of two types of energy harvesters was evaluated by the vibration system described above for the dynamic piezoelectric coefficient measurements. The results illustrated the potential of the concentric tunnel FEP films for different working circumstances. Output power was obtained for comparison with other ferroelectret harvesters, which can be calculated by

(7) $$P_{out}=\frac{U_{rms}^{2}}{R_{l}}=R_{l}I_{rms}{}^{2}=R_{l}\omega^{2}Q_{rms}^{2}$$

where *R_l_*, *ω*, *U_rms_*, *I_rms_*, and *Q_rms_* are the load resistance, operating angular frequency of the energy harvesting system, root mean square (rms) value of voltage, current, and rms charge, respectively.

At resonance angular frequency, *ω*_0_, and match resistance, the output power is maximal and can be determined by

(8) $$P_{out}=R_{l}I^{2}=\frac{1}{2}R_{opt}\omega_{0}{}^{2}Q_{SC}^{2}$$

where *Q_SC_* is the rms value of charge measured in short-circuit and *R_opt_* is the optimal load resistance at the resonance frequency, which can be expressed by

(9) $$R_{opt}=\frac{1}{\omega_{0}C}$$

where *C* represents the capacitance of the sample.

**Figure 13:**
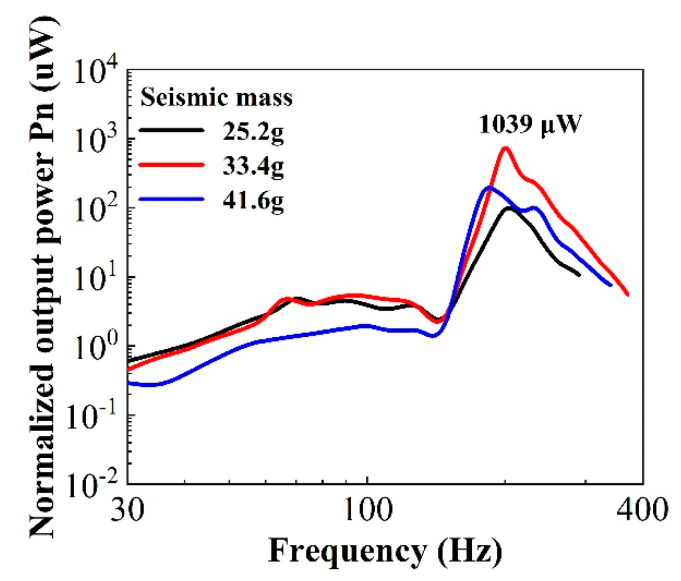
Normalized output power experimental data of the 33-mode energy harvester.

The normalized output power as a function of vibration frequency for a 33-mode harvester under different seismic masses at an optimal load resistance is shown in Figure 13. The sample had a diameter of 20 mm and capacitance of 63.5 pF, and the seismic masses were 25.5, 33.4, and 41.6 g with the same diameter as the sample, respectively. As can be seen, the resonance frequency changed with the seismic mass. Hence, different optimal load resistances were determined using Equation (9). The figure indicates that the maximum power of the 33-mode harvester was about 1039 μW with the seismic mass of 33.4 g and optimal load resistance of 12 MΩ at the resonance frequency of 200 Hz.

**Figure 14:**
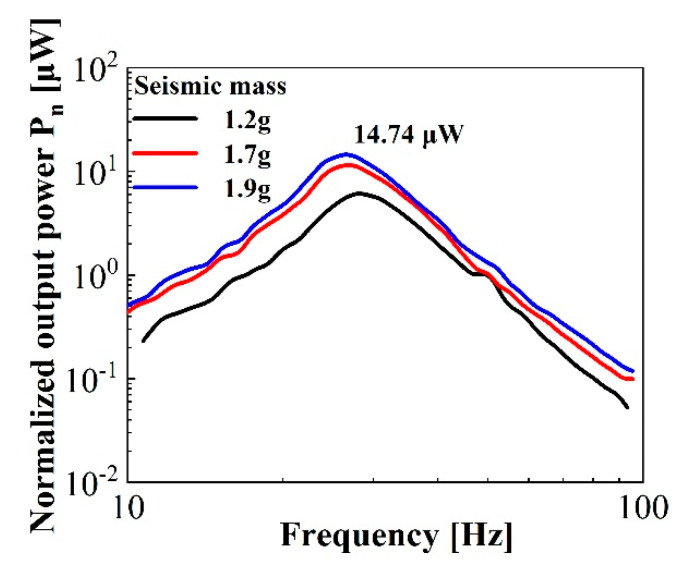
Normalized output power experimental data of the 31-mode energy harvester.

The output performance of the 31-mode harvester was investigated later. Figure 14 gives the experimental data of a representative sample with a diameter of 30 mm. The seismic masses with a diameter of 8 mm were placed in the centermost part of the sample in order to maximize the radial piezoelectric response. As shown in the figure, the resonance frequencies and normalized output power also changed with increasing seismic mass. With the example of the curve with the seismic mass of 1.9 g, marked in blue, given *C* = 176.8 pF and *f_0_* = 26.3 Hz, the optimal load resistance *R*_opt_ was calculated as 34 MΩ and the maximum output power was determined as approximately 15 μW following Equation (8).

In order to compare the performance of the 33-mode and 31-mode energy harvesters with other small scale harvesters, the area power density was defined as *P_a_* = *P_m_*/*A*, where *P_m_* is the maximum power generated by the harvester at the resonance frequency, optimal resistance, and appropriate seismic mass, and *A* is the active area of the harvester. Substituting the experimental values of *P_m_* = 1039 and 15 μW and *A* = 3.14 and 7.07 cm^2^ for the 33- and 31-mode devices, respectively, the area power densities are 331 μW/cm^2^ and 2.12 μW/cm^2^, respectively. As shown in Table 1, the area power density of the 33-mode harvester exceeds that of other nanogenerators using other materials, such as PVDF, PDMS, and IXPP, and is close to the power density generated by the parallel tunnel structure FEP ferroelectrets. However, it should be kept in mind that the appropriate load mass of 33.4 g was applied to a sample with an area of 3.14 cm^2^, corresponding to a pressure of 1 kPa, which is larger than the load pressure in other studies. It proves that the FEP ferroelectret films with a tunnel structure are excellent piezoelectric transduction materials for energy harvesting.

For the 31-mode harvester, however, the area power density was two orders of magnitude lower than the parallel tunnel structure FEP films. The reason for this is that when PCDs are subjected to annular clamping, the boundary region has a very small effective strain and the amplitude of the center part is also small. At the same time, it is difficult for air in the enclosed concentric tunnels to be released during vibration, especially at low frequencies, which will affect the deformation of the tunnels. It is expected that by designing new shapes of concentric tunnel structures, such as a complete disc cut into propeller shapes, the output power can be significantly increased. Relevant work is underway.

## 4. Summary and Discussion

In conclusion, FEP bipolar ferroelectret films with a specifically designed concentric tunnel structure were prepared by means of rigid template-based thermoplastic molding and contact polarization. The fabricated films featured both remarkable longitudinal and radial piezoelectric activities, as well as superior thermal stability. For the longitudinal piezoelectric activity, a quasi-static piezoelectric *d*_33_ coefficient of up to 5280 pC/N was achieved. For the radial piezoelectric activity, the piezoelectric sensitivity, *M*_r_, of 40,000 pC/N was obtained in a sample with a diameter of 30 mm at a static force of 200 mN.

For energy harvesting with such concentric tunnel FEP ferroelectret films, power output of up to 1 mW was achieved in an energy harvester working in 33-mode and at 210 Hz, referring to a seismic mass of 33.4 g and an acceleration of 1 *g*. For a device working in 31-mode, the power output of 15 μW was obtained at a relatively small resonance frequency of 26 Hz and a very light seismic mass of 1.9 g. Besides, our experimental results on the fatigue resistance (not shown here) show that the stable output power of the devices was around 90% after more than 1 million operation cycles. Therefore, depending on specific circumstances, such films provide flexible options for designing vibrational energy harvesters working either in 33-mode or 31-mode.

## Figures and Tables

**Table 1:** Recent progress of other small-scale harvesters.

Type	Area Power Density	Pressure or Seismic Mass	References
PVDF/KNN/ZnO electrospun nanofibers	11.31 μW/cm^2^	~1 kPa	[43]
PVDF/BT nanoparticles	120 μW/cm^2^	----	[44]
ZnSnO_3_ nanowires/PDMS	230 μW/cm^2^	>100 N	[45]
MASnBr_3_/PDMS	74.52 μW/cm^2^	~0.5 MPa	[46]
IXPP ferroelectret (33-mode)	57.32 μW/cm^2^	33.7 g	[47]
FEP ferroelectret (out-of-plane type)	150 μW/cm^2^	10 g	[48]
Cross-tunnel FEP ferroelectret (33-mode)	0.12 μW/cm^2^	69.5 g	[49]
Parallel tunnel FEP ferroelectret (33-mode)	462.5 μW/cm^2^	80 g	[50]
Parallel tunnel FEP ferroelectret (31-mode)	95.83 μW/cm^2^	2 g	[28]
Parallel tunnel FEP ferroelectret (31-mode) advanced design	272.5 μW/cm^2^	0.3 g	[22]
Cantilever-based parallel tunnel FEP ferroelectret (31-mode)	106.7 μW/cm^2^	4.5 g	[29]
Concentric tunnel FEP ferroelectret (33-mode)	331 μW/cm^2^	33.4 g	This work
Concentric tunnel FEP ferroelectret (31-mode)	2.12 μW/cm^2^	2.0 g	This work
